# Comparison of discriminative motif optimization using matrix and DNA shape-based models

**DOI:** 10.1186/s12859-018-2104-7

**Published:** 2018-03-06

**Authors:** Shuxiang Ruan, Gary D. Stormo

**Affiliations:** Department of Genetics and Edison Family Center for Genome Sciences and Systems Biology, Washington University School of Medicine, St. Louis, 63110 USA

**Keywords:** Motif, Motif optimization, ChIP-seq, Position weight matrix, DNA shape features

## Abstract

**Background:**

Transcription factor (TF) binding site specificity is commonly represented by some form of matrix model in which the positions in the binding site are assumed to contribute independently to the site’s activity. The independence assumption is known to be an approximation, often a good one but sometimes poor. Alternative approaches have been developed that use *k*-mers (DNA “words” of length *k*) to account for the non-independence, and more recently DNA structural parameters have been incorporated into the models. ChIP-seq data are often used to assess the discriminatory power of motifs and to compare different models. However, to measure the improvement due to using more complex models, one must compare to optimized matrix models.

**Results:**

We describe a program “Discriminative Additive Model Optimization” (DAMO) that uses positive and negative examples, as in ChIP-seq data, and finds the additive position weight matrix (PWM) that maximizes the Area Under the Receiver Operating Characteristic Curve (AUROC). We compare to a recent study where structural parameters, serving as features in a gradient boosting classifier algorithm, are shown to improve the AUROC over JASPAR position frequency matrices (PFMs). In agreement with the previous results, we find that adding structural parameters gives the largest improvement, but most of the gain can be obtained by an optimized PWM and nearly all of the gain can be obtained with a di-nucleotide extension to the PWM.

**Conclusion:**

To appropriately compare different models for TF bind sites, optimized models must be used. PWMs and their extensions are good representations of binding specificity for most TFs, and more complex models, including the incorporation of DNA shape features and gradient boosting classifiers, provide only moderate improvements for a few TFs.

**Electronic supplementary material:**

The online version of this article (10.1186/s12859-018-2104-7) contains supplementary material, which is available to authorized users.

## Background

The interaction between proteins and genomic DNA plays a crucial role in many important cellular processes. For instance, the RNA polymerase interacts with DNA during transcription and uses it as a template for RNA synthesis [1] and the formation of nucleosomes involves histones and DNA binding together to form a well-defined three-dimensional structure [2]. Some epigenetic modifications such as DNA methylation, which alter DNA accessibility and chromatin structures, are carried out by the DNA methyltransferase and other proteins that mainly target CpG di-nucleotides [3]. The sequence-specific transcription factors (TFs) are a special class of DNA-binding proteins that recognize specific DNA sequences and primarily regulate gene expression [4, 5]. In most species, they constitute between 5% and 10% of all genes [6–8]. Although some prominent TFs, including Sox [9], AP-1 [10, 11] and Sp1 [12], have been studied extensively, the binding specificities of most TFs are poorly documented even in many well-studied species [13]. In recent years, several high-throughput experimental techniques, such as high-throughput SELEX (HT-SELEX), protein-binding microarrays (PBMs) and ChIP-seq, have been developed to estimate the relative binding affinities of large numbers of DNA sequences both in vitro and in vivo [14–17]. These techniques have greatly accelerated the study of TF binding specificity [4], but the analysis of their results proves challenging and requires the development of novel TF binding models and motif discovery algorithms.

The specificity of TFs is commonly represented by matrix models, of which there are several varieties [18]. In probabilistic models, such as position frequency matrices (PFMs), the matrix elements are the probability of each base occurring at each position in the binding site and the probability of a specific site is the product of those probabilities for the base at each position. In a more general position weight matrix (PWM), the elements of the matrix are added together to get the score for a specific binding site. Trained on quantitative binding data, regression methods can be used to obtain matrix elements that correspond to energy contributions of each base at each position [19–21]. All matrix models have in common the assumption that the positions of a binding site contribute independently to its activity, an assumption that is often a good approximation but not always [22–24]. More complex models utilize *k*-mers, short DNA words of length *k*, to account for non-independence between positions [21, 22, 25–27]. Recently there have been several studies showing that variations in DNA shape can influence TF binding affinity, and that those contributions may involve non-independence between positions [28–31]. DNAshape is a program that predicts DNA structural features in a high-throughput manner based on Monte Carlo simulations of DNA fragments [32]. The Genome Browser for DNA shape annotations (GBshape), a database based on DNAshape and related computational tools, provides DNA shape feature predictions for a range of organisms [33]. Those resources were used in a recent study where motif models using gradient boosting classifiers were trained to differentiate ChIP-seq peaks from random background sequences, showing that adding DNA shape features can significantly improve the accuracy of the classifiers [34].

In this report, we replicate the results of Mathelier et al. [34] and we compare the performance of the gradient boosting classifiers to simple PWMs generated by DAMO, a perceptron-based optimization method that finds the optimal PWM with the highest area under the receiver operating characteristic curve (AUROC). DAMO is similar to our previously described DiMO [35], but where DiMO provided optimized PFMs, DAMO provides optimized PWMs which have recently been shown to avoid the inherent limitations of probabilistic models [36]. DAMO also allows for the inclusion of adjacent di-nucleotides if the independence assumption provides poor performance. Our results confirm that adding DNA shape features in a gradient boosting classifier does significantly improve the performance over the initial JASPAR PFMs, but also show that most of the improvement can be obtained with optimal PWMs, and adding di-nucleotide contributions performs nearly as well as the much more complex gradient boosting classifiers including shape parameters.

## Methods

### JASPAR PFMs

Following the study of Mathelier et al. [34], we obtained 75 JASPAR PFMs that can be associated with ChIP-seq datasets generated by the ENCODE project [37]. (Their work included 76 PFMs but one of those (ID: MA0133.1) is no longer available in the March 2017 JASPAR CORE collection).

### ChIP-seq datasets

We used the same ChIP-seq datasets analyzed by Mathelier et al. [34] and downloaded 396 uniformly processed human ENCODE ChIP-seq datasets associated with the 75 JASPAR PFMs from the UCSC Genome Browser [38]. For each ChIP-seq peak, we retrieved the 100 bp sequence centered on the point-source of the peak from the human genome assembly hg19, which serves as a positive sequence for training and testing TF binding models. For each positive sequence, we also constructed a negative sequence, which is the 100 bp sequence 100 bp downstream from the positive sequence in the human genome. We also tested performance when the negative sequences were obtained from 5000 bp downstream. For each ChIP-seq dataset, we constructed 10 training and 10 testing sets for 10-fold cross-validation, where each training set is 9 times the size of a testing set. We also tested performance when the training set and testing set were each only 10% of the total data.

### DNA shape features

We retrieved the same DNA shape features used by Mathelier et al. [34] from GBshape [33]. The features include the helix twist (HelT), the minor groove width (MGW), the propeller twist (ProT), the roll (Roll), and the corresponding second-order shape features. These features were only used for training and testing models designated with “+ shape”.

### Motif optimization algorithms evaluated

Table 1 lists the motif optimization algorithms evaluated in this study. Of the 9 algorithms, 5 are based on the DNAshapedTFBS program, which trains a binary classifier using the gradient boosting algorithm [34]. These 5 algorithms differ in two aspects: 1) how the feature vector is encoded, and 2) whether the feature vector includes DNA shape features. In the 4bit encoding, which was used by Mathelier et al., A is encoded as 1000, T as 0100, G as 0010, and C as 0001. In JASPAR + shape and DAMO + shape the sequence information is included simply as a score from a matrix model and in Shape_only it is not included at all. The remaining 4 algorithms are simple matrix models from the single nucleotide and adjacent di-nucleotide mode of the DAMO program, the JASPAR PFMs and PFMs obtained from the DAMO PWMs (which are equivalent to the original DiMO PFMs). DAMO is a Python implementation of the DiMO program, which is based on perceptron learning and finds the optimal PWM by maximizing its AUROC score [35]. The original DiMO program outputs a normalized PFM derived from the optimal PWM, even though it uses a PWM internally for optimization. Because PWMs do not have the limitations of probabilistic PFMs [36], we configured the DAMO program to output the optimal PWM directly and we also allow for adjacent di-nucleotides to be included to capture non-independent contributions from adjacent positions in the binding sites [21, 27].

**Table 1:** Descriptions of the motif optimization algorithms evaluated

Algorithm	Output	Description
JASPAR	Position frequency matrix	PFMs from the JASPAR database
DAMO	Position weight matrix	Modified DiMO program that outputs PWMs instead of PFMs
DAMO_PFM	Position frequency matrix	PFMs derived from the DAMO single-nucleotide PWMs
DAMO_dinuc	Position weight matrix	The adjacent di-nucleotide mode of DAMO
DNAshapedTFBS_4bit	Gradient boosting classifier	DNAshapedTFBS with 4-bit encoding
DNAshapedTFBS_4bit + shape	Gradient boosting classifier	DNAshapedTFBS_4bit plus DNA shape features
Shape_only	Gradient boosting classifier	The feature vector contains only DNA shape features
JASPAR + shape	Gradient boosting classifier	JASPAR PFM score plus DNA shape features
DAMO + shape	Gradient boosting classifier	DAMO single-nucleotide PWM score plus DNA shape features

### Training and testing binding models

The training and testing procedures are based on the methods described by Mathelier et al. [34].

#### Preprocessing

We first use the JASPAR PFM to scan all the positive and negative sequences in both the training and testing set, and identify the best binding site, which has the same length as the PFM, within each sequence. Then we use the DNAshapedTFBS program to extract the DNA sequence of each best site and the corresponding DNA shape features. The sequences of the best sites, instead of the full-length positive and negative sequences, are used in the following steps for training and testing TF binding models. This means that we are not testing motif discovery algorithms, because the positive and negative sites are predefined. Rather, we are testing how well models of different complexity can perform classification after optimization for that task (except for the original JASPAR PFMs).

#### Training

The training procedure depends on the motif optimization algorithm. For the DNAshapedTFBS-based methods, we first construct, for each best site in the training set, a feature vector containing its JASPAR PFM score, the DAMO PWM score or encoded DNA sequence. If the method takes account of the DNA shape features, the feature vector also contains the normalized values of the 8 DNA shape features at each position. Then a gradient boosting classifier is trained on the positive and negative feature vectors. For DAMO, the sequences of the positive and negative sites are directly fed into the program along with the JASPAR PFM, which serves as a seed matrix. DAMO then finds the optimal PWM that maximizes the AUROC on the training set, using the perceptron training algorithm. The perceptron training, described in detail previously [35], updates the PWM by error correction on the mis-classified sites, those in the positive set with lower scores than the best negative site, and the negative sites with scores higher than the lowest positive site, and training proceeds until convergence. The sequences can be encoded using adjacent dinucleotides to capture non-independent contributions between those positions [18, 21, 27]. The DAMO_PFM model is obtained by considering the DAMO PWMs scores as energies and converting to normalized probabilities (as in the original DiMO approach [35]).

#### Testing

The testing procedure is the same for all the algorithms. The trained gradient boosting classifiers and the different PFM and PWM models are used to score all the positive and negative sites in the testing set. Those scores are used to rank the sites and compute the area under the precision recall curve (AUPRC) and the area under the receiver operating characteristic curve (AUROC) based on the true labels of the ranked sites. We report the mean values and standard deviations from ten-fold cross-validation tests.

## Results

For each algorithm, the mean and standard deviation of the AUPRC scores for the 396 samples, on both training and testing sets, are summarized in Table 2. (AUROC scores are reported in Additional file 1: Table S1.) As reported previously, adding shape parameters to the JASPAR PFMs significantly improves the AUPRC [34]. We obtain a mean increase of 0.034, equivalent to adding shape parameters to the DAMO scores, and larger than the improvement of any other model. But just optimizing the PFM, with the DAMO_PFM model, captures about 35% of the total improvement. The PWM obtained by DAMO provides nearly 60% of the total improvement and demonstrates the inherent advantage of a PWM model over a PFM model as we showed previously [36]. Adding parameters for adjacent di-nucleotides captures nearly 80% of the improvement over the JASPAR PFM model. All of those models are simple matrix models where the positions contribute independently to the total score of a site, except that in the DAMO_dinuc model the adjacent dinucleotides contribute additively to the score. The gradient boosting classifiers, which use an ensemble of decision trees for classification, are complex non-linear models even without the shape parameters. The 4bit model, whose input is only the sequence, increases performance to 88% of the best model and adding shape parameters to the 4bit classifier increases performance to essentially the same as JASPAR + shape and DAMO + shape. Interestingly, the Shape_only model does nearly as well as any other gradient boosting classifier model, indicating that the shape parameters inherently contain the sequence information (see Discussion). We also tested performance when the negative sequence set was selected at a distance of 5000 bp instead of 100 bp downstream (Additional file 2: Tables S2). In that case the performance on both the training and testing sets was increased, probably because sequences in the negative set that are only 100 bp downstream of the ChIP-seq peak may also contain true binding sites. But the overall results are consistent with the initial findings. While the increase in AUPRC of JASPAR + shape over JASPAR PFMs is now 0.065, nearly 70% of that increase is captured using the DAMO PWMs, and nearly 90% is captured by the DAMO_dinuc model.

**Table 2:** Mean AUPRC (and standard deviation) on ChIP-seq data

Algorithm	Training	Testing
JASPAR	0.812 (0.132)	0.812 (0.132)
DAMO	0.834 (0.119)	0.832 (0.120)
DAMO_PFM	0.825 (0.120)	0.824 (0.122)
DAMO_dinuc	0.844 (0.114)	0.839 (0.119)
DNAshapedTFBS_4bit	0.854 (0.105)	0.842 (0.115)
DNAshapedTFBS_4bit + shape	0.875 (0.090)	0.845 (0.113)
Shape_only	0.871 (0.089)	0.840 (0.112)
JASPAR + shape	0.878 (0.089)	0.846 (0.112)
DAMO + shape	0.879 (0.090)	0.846 (0.113)

**Fig. 1:**
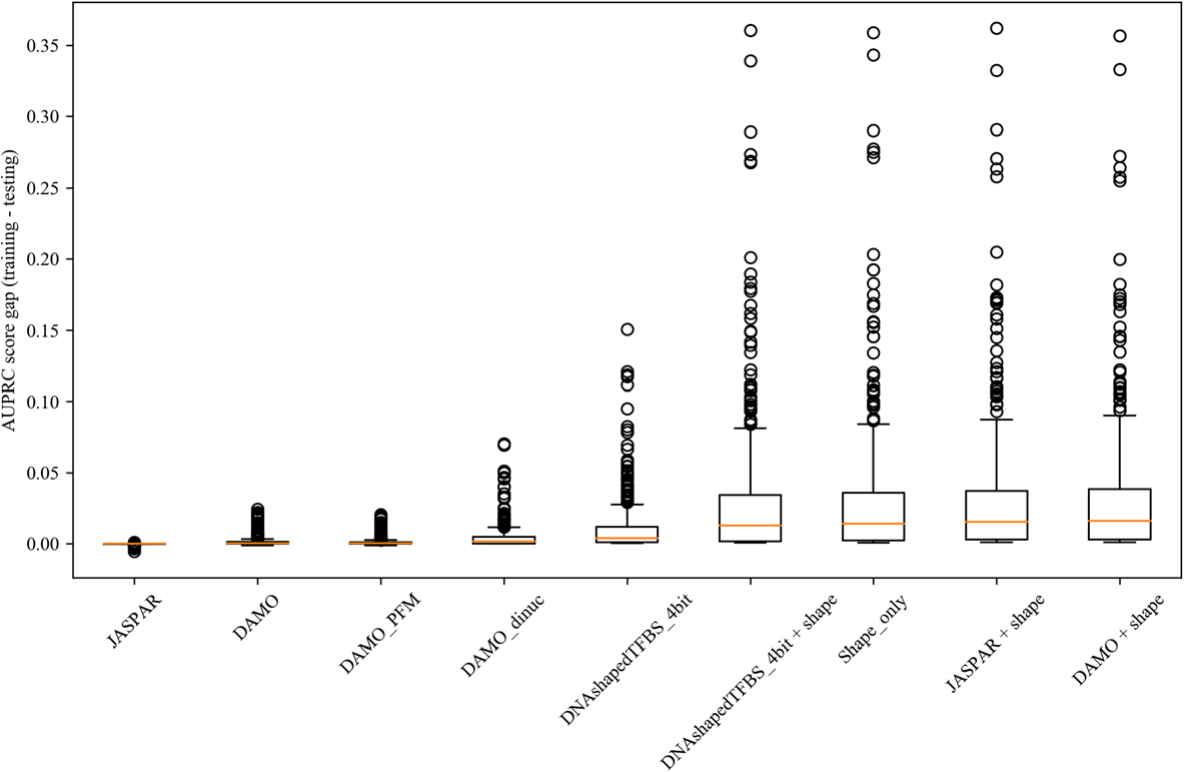
Differences in AUPRC between training and testing datasets. For each model the differences are shown for each of the 396 datasets. The box represents 1st, 2nd (median indicated with line) and 3rd quartiles and the whiskers represent 1.5 interquartile range (IQR) below or above 1st or 3rd quartiles

The gap between the training and testing scores is a measure of overfitting for a model, which generally corresponds to the complexity of the model. Specifically, the highly non-linear DNAshapedTFBS-based models, with their ensemble of decision trees, have larger gaps than the PFM and PWM models, which are linear models (Fig. 1). In fact, the training and testing scores of the linear models are nearly identical. The largest gaps, > 0.03, are associated with the DNAshapedTFBS-based models with DNA shape features, presumably because their feature vectors are most complex. This effect also shows up in the sensitivity of the complex models to the size of the training data. Additional file 3: Table S3 shows results for several of the models when trained on only 1/10 of the data, the same as the testing sample size, and much smaller than the normal training on 9/10 of the data. All of the models, except for the JASPAR PFMs which are untrained, increase the AUPRC and AUROC scores on the training data and decrease those scores on the testing data. On those small training sets the DAMO PWMs score as well as the more complex models on the testing sets.

**Fig. 2:**
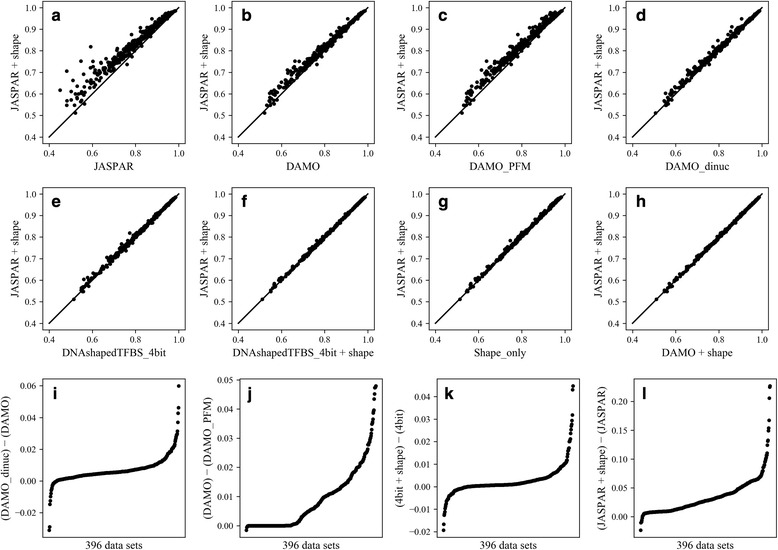
Comparison of AUPRC scores for different models. **a**-**h** JASPAR + shape on vertical axis and each of the other eight models on the horizontal axis. **i** Difference in AUPRC for DAMO PWM with and without di-nucleotides. **j** Difference in AUPRC for DAMO PWM and DAMO PFM. **k**-**l** Differences with adding shape features to the 4bit model and the JASPAR PFM model

Figure 2 compares graphically the results reported in Table 2, with points for each of the 396 ChIP-seq datasets. The top eight panels have the JASPAR + shape AUPRC results on the vertical axis and each of the other eight models on the horizontal axis. Consistent with Table 2, there is a large improvement from the JASPAR scores alone (Fig. 2a). For the DAMO PWMs (Fig. 2b) there are many fewer data sets with large improvement. The DAMO PFMs are much better than the JASPAR PFMs, as expected because those JASPAR PFMs have not been optimized for this task, but are not as good as the DAMO PWMs showing the inherent limitations of PFM models [36]. The DAMO_dinuc model (Fig. 2d) has very few datasets with large improvements. In each of the other models, which come from the gradient boosting classifier (Fig. 2e-h), the data points cluster near the diagonal, indicating that the difference between the two scores of the same sample is very small. The bottom row of panels in Fig. 2 show the score differences, in ascending order, between similar models. Note the differences in scale on the vertical axes. In most cases there are very few datasets with differences > 0.02, except for the comparison of the JASPAR scores and JASPAR + shape, where many datasets show improvements > 0.05 and a few are > 0.10. Most TFs have multiple associated ChIP-seq data sets (median of three), and the mean difference for every TF are shown in Additional file 4: Table S4. Except for the comparison of JASPAR + shape with JASPAR, very few of the TFs have mean differences > 0.02, suggesting that feature vectors based only on sequence, optimized for AUROC scores but without including structure parameters, capture essentially all of the discriminatory power of the motifs. However, the motifs using the ensemble of decision trees do contain higher-order information beyond that available to simple PWMs, most of which is captured by using di-nucleotide extensions of PWMs.

## Discussion

Our results confirm that adding DNA shape features significantly improves the performance of JASPAR PFMs, with a mean increase of 0.034 in AUPRC. Simply optimizing the PFMs for the task of maximizing AUROC captures over one-third of that difference. An optimized PWM captures most of the improvement, and adding di-nucleotide parameters helps further. The gradient boosting approach increased AUPRC slightly more, as did adding shape parameters, but on the vast majority of datasets the differences between the simple PWM models and more complex models are small, consistent with previous work showing that optimized PWMs are often good approximations for TF specificity [22–24]. Including DNA shape features further increases the number of parameters in a binding model, which increases the cost of training and may result in overfitting. The fact that the performance of DAMO_dinuc is similar to the non-linear gradient boosting classifiers indicates that the majority of the deviations from the assumption of position independence can be captured by adjacent di-nucleotide interactions.

The success of the PWMs does not mean that the structure of DNA plays no role in binding site recognition. In fact, there are good examples showing that it does [28–30, 39]. All of the models based on sequence features alone are agnostic with respect to the mechanisms of specificity. They only describe mathematically how much each base at each position contributes to binding specificity, or in the case of higher-order contributions, how useful those are in discriminating the positive and negative training sets. Because DNA structure depends on sequence, redundancies arise when using both types of parameters together. In fact, given a sufficiently long sequence (such as a genome) encoded solely with structure parameters, a good compression algorithm could reconstruct the sequence exactly, demonstrating that the structure information contains within it the sequence information. This is also clear from our results with the Shape_only model. Certainly interactions between the TF and the bases of the DNA sequence are the primary contributions to binding affinity. But encoding the sequence using only structural parameters performs nearly as well as using input vectors including both sequence and structure because the sequence is redundant given the structure.

We advocate using the most efficient algorithm, with the least number of parameters, that obtains the maximum fit to quantitative data, or the optimal discrimination between positive and negative data sets. This reduces the complexity of the model to only the non-redundant parameters, minimizes the training time and reduces the susceptibility to over-fitting. Those optimal parameters, including higher-order interactions as needed, can be used to infer the mechanism of binding. For example, if dinucleotides are required to obtain the best fit, and the specific dinucleotides that correspond to higher affinity (or better discrimination) are those correlated with a narrow minor groove, then one could infer the TF prefers binding to DNA structures with narrow minor grooves. But doing this after the mathematically optimal parameters are obtained removes redundancies in the feature vectors used for training which could confound interpretation.

Discrimination of binding sites from ChIP-seq data, such as with AUPRC or AUROC scores, is a popular method for assessing the accuracy of TF motifs [40]. However, those scores are inherently rank based and miss other important aspects of binding activity such as the relative binding affinity between different binding sites [17, 20]. Therefore PWMs, and other motifs, obtained simply by maximizing AUPRC or AUROC scores should not be used as predictors of relative binding affinity. To do that they should be rescaled by reference to some external binding data, preferably from quantitative in vitro experiments. Alternatively, one can assume that the majority of peaks contain binding sites within some constrained range of binding affinity, perhaps within 100-fold of the maximum, and use that assumption to scale the PWM to approximate binding energies [20].

## Conclusions

To address the issue of whether matrix models, which assume independent contributions across the positions of the binding site, are adequate representations of specificity requires appropriate comparisons. To compare complex models that have been optimized for a specific task, such as maximizing AUROC, to PFM/PWM models that have been obtained from other types of data or for other tasks, confounds the comparison between the type of model and the method for obtaining the model parameters. We show that simple PWM models, when optimized for maximum AUROC, perform nearly as well as more complex non-linear models. We also show the advantages of PWMs over PFMs, and that including adjacent dinucleotides in the additive PWM model can further enhance its performance on at least some of the datasets. While DNA structure certainly contributes to binding affinity, at least in some cases, we advocate for finding mathematically optimal models that are simple and efficient but agnostic as to mechanism, and then inferring the mechanisms that contribute to binding affinity as further steps in the analysis.

## Additional files

Additional file 1: **Table S1.** Mean AUROC (and standard deviation) on ChIP-seq data. (DOCX 13 kb)

Additional file 2: **Table S2.** Effect of Method for Generating Negative Sequences on Training and Testing Scores. (DOCX 13 kb)

Additional file 3: **Table S3.** Scores for the motif optimization algorithms on ChIP-seq data with small training sets. (DOCX 12 kb)

Additional file 4: **Table S4.** AUPRC and AUROC differences between model pairs by TF. (XLSX 32 kb)
