# Supplementary material for: Comparison of discriminative motif optimization using matrix and DNA shape-based models
Source: BMC Bioinformatics. 2018 Mar 6;19:86. doi: 10.1186/s12859-018-2104-7 (PMC5840810; doi:10.1186/s12859-018-2104-7)
Supplement: Supplementary file 1 — Table S1. Mean AUROC (and standard deviation) on ChIP-seq data. (DOCX 13 kb) [file 12859_2018_2104_MOESM1_ESM.docx]

Table S1. Mean AUROC (and standard deviation) on ChIP-seq data

| Algorithm | Training | Testing |
| --- | --- | --- |
| JASPAR | 0.788 (0.139) | 0.788 (0.139) |
| DAMO | 0.817 (0.120) | 0.815 (0.122) |
| DAMO_PFM | 0.807 (0.122) | 0.806 (0.123) |
| DAMO_dinuc | 0.829 (0.115) | 0.823 (0.120) |
| DNAshapedTFBS_4bit | 0.841 (0.105) | 0.826 (0.117) |
| DNAshapedTFBS_4bit + shape | 0.867 (0.089) | 0.828 (0.115) |
| Shape_only | 0.863 (0.088) | 0.823 (0.114) |
| JASPAR + shape | 0.870 (0.089) | 0.830 (0.115) |
| DAMO + shape | 0.871 (0.090) | 0.831 (0.115) |
