# Supplementary material for: Comparison of discriminative motif optimization using matrix and DNA shape-based models
Source: BMC Bioinformatics. 2018 Mar 6;19:86. doi: 10.1186/s12859-018-2104-7 (PMC5840810; doi:10.1186/s12859-018-2104-7)
Supplement: Supplementary file 2 — Table S2. Effect of Method for Generating Negative Sequences on Training and Testing Scores. (DOCX 13 kb) [file 12859_2018_2104_MOESM2_ESM.docx]

Supplementary Tables S2. Effect of Method for Generating Negative Sequences on Training and Testing Scores

In this supplementary information, we change the method for generating negative sequences for both the training and testing datasets and report the corresponding scores. In the main text, the negative sequence is the 100 bp sequence 100 bp downstream from each positive sequence (ChIP-seq peak) in the human genome. To investigate the effect of the method for generating negative sequences on training and testing scores, we generated another set of negative sequences which are 100 bp sequence 5000 bp downstream from each positive sequence. The two tables below list the new training and testing scores (AUPRC and AUROC scores separately) for 5 algorithms: JASPAR, DAMO, DAMO_dinuc, DNAshapedTFBS_4bit and JASPAR + shape. These algorithms should be a good representation of all the different algorithms evaluated in the main text.

Table S2.1. Mean AUPRC (and standard deviation) on ChIP-seq data

| Algorithm | Training | Testing |
| --- | --- | --- |
| JASPAR | 0.821 (0.135) | 0.821 (0.135) |
| DAMO | 0.867 (0.097) | 0.866 (0.098) |
| DAMO_dinuc | 0.881 (0.089) | 0.878 (0.092) |
| DNAshapedTFBS_4bit | 0.890 (0.081) | 0.882 (0.087) |
| JASPAR + shape | 0.908 (0.071) | 0.886 (0.085) |

Table S2.2. Mean AUROC (and standard deviation) on ChIP-seq data

| Algorithm | Training | Testing |
| --- | --- | --- |
| JASPAR | 0.794 (0.151) | 0.794 (0.151) |
| DAMO | 0.856 (0.097) | 0.855 (0.098) |
| DAMO_dinuc | 0.871 (0.090) | 0.867 (0.093) |
| DNAshapedTFBS_4bit | 0.882 (0.081) | 0.873 (0.087) |
| JASPAR + shape | 0.903 (0.070) | 0.877 (0.085) |
