# Supplementary material for: Comparison of discriminative motif optimization using matrix and DNA shape-based models
Source: BMC Bioinformatics. 2018 Mar 6;19:86. doi: 10.1186/s12859-018-2104-7 (PMC5840810; doi:10.1186/s12859-018-2104-7)
Supplement: Supplementary file 3 — Table S3. Scores for the motif optimization algorithms on ChIP-seq data with small training sets. (DOCX 12 kb) [file 12859_2018_2104_MOESM3_ESM.docx]

Table S2 Scores for the motif optimization algorithms on ChIP-seq data with small training sets

| Algorithm | Training AUPRC | Training AUROC | Testing AUPRC | Testing AUROC |
| --- | --- | --- | --- | --- |
| DAMO | 0.842 (0.112) | 0.828 (0.112) | 0.829 (0.123) | 0.812 (0.126) |
| JASPAR | 0.812 (0.132) | 0.788 (0.139) | 0.813 (0.131) | 0.788 (0.140) |
| DNAshapedTFBS_4bit | 0.896 (0.083) | 0.892 (0.083) | 0.829 (0.126) | 0.813 (0.128) |
| JASPAR + shape | 0.951 (0.051) | 0.951 (0.050) | 0.829 (0.124) | 0.811 (0.128) |
| DNAshapedTFBS_4bit + shape | 0.948 (0.051) | 0.949 (0.050) | 0.824 (0.128) | 0.807 (0.130) |

This table is based on training sets of 1/10 of the data, the same size as the testing sets.
